# Feed Restriction Modulates the Fecal Microbiota Composition, Nutrient Retention, and Feed Efficiency in Chickens Divergent in Residual Feed Intake

**DOI:** 10.3389/fmicb.2018.02698

**Published:** 2018-11-19

**Authors:** Sina-Catherine Siegerstetter, Renée M. Petri, Elizabeth Magowan, Peadar G. Lawlor, Qendrim Zebeli, Niamh E. O'Connell, Barbara U. Metzler-Zebeli

**Affiliations:** ^1^Institute of Animal Nutrition and Functional Plant Compounds, Department for Farm Animals and Veterinary Public Health, University of Veterinary Medicine Vienna, Vienna, Austria; ^2^Agriculture Branch, Agri-Food and Biosciences Institute, Hillsborough, United Kingdom; ^3^Teagasc, Pig Development Department, Animal & Grassland Research & Innovation Centre, Moorepark, Ireland; ^4^Institute for Global Food Security, Queen's University Belfast, Belfast, United Kingdom

**Keywords:** broiler chicken, fecal microbiota, feed intake level, nutrient retention, residual feed intake

## Abstract

There is a great interest to understand the impact of the gut microbiota on host's nutrient use and FE in chicken production. Both chicken's feed intake and gut bacterial microbiota differ between high and low-feed efficient chickens. To evaluate the impact of the feed intake level on the feed efficiency (FE)-associated variation in the chicken intestinal microbiota, differently feed efficient chickens need to eat the same amount of feed, which can be achieved by feeding chickens restrictively. Therefore, we investigated the effect of restrictive vs. *ad libitum* feeding on the fecal microbiome at 16 and 29 days posthatch (dph), FE and nutrient retention in chickens of low and high residual feed intake (RFI; metric for FE). Restrictively fed chickens were provided the same amount of feed which corresponded to 85% of the *ad libitum* fed group from 9 dph. FE was determined for the period between 9 and 30 dph and feces for nutrient retention were collected on 31 to 32 dph. From the 112 chickens (*n* = 56 fed *ad libitum*, and *n* = 56 fed restrictively), 14 low RFI and 15 high RFI *ad libitum* fed chickens, and 14 low RFI (*n* = 7 per sex) and 14 high RFI restrictively fed chickens were selected as the extremes in RFI and were retrospectively chosen for data analysis. Bray-Curtis dissimilarity matrices showed significant separation between time points, and feeding level groups at 29 dph for the fecal bacterial communities. Relevance networking indicated positive associations between *Acinetobacter* and feed intake at 16 dph, whereas at 29 dph *Escherichia*/*Shigella* and *Turicibacter* positively and *Lactobacillus* negatively correlated to chicken's feed intake. *Enterobacteriaceae* was indicative for low RFI at 16 dph, whereas *Acinetobacter* was linked to high RFI across time points. However, restrictive feeding-associated changes in the fecal microbiota were not similar in low and high RFI chickens, which may have been related to the higher nutrient retention and thus lower fecal nutrient availability in restrictively fed high RFI chickens. This may also explain the decreased RFI value in restrictively fed high RFI chickens indicating improved FE, with a stronger effect in females.

## Introduction

Finding effective strategies to improve chicken's feed efficiency (FE) is a major goal to decrease overall production costs, preserve additional edible resources for humans, and reduce the ecological footprint of chicken rearing systems (Bottje and Carstens, 2009; Yan et al., 2017). Chickens of divergent FE differ in several phenotypic characteristics with the most obvious difference being the lower feed intake of high feed-efficient chickens compared to low feed-efficient chickens (Siegerstetter et al., 2017, 2018). In addition, FE-related variation in the intestinal microbiota of chickens has been reported, indicating that the intestinal microbiota may have modified the utilization of dietary nutrients by the host (Crisol-Martínez et al., 2017; Siegerstetter et al., 2017, 2018). In general, chicken's feed intake level can have a profound impact on digesta volume, retention time, and nutrient digestion, thereby affecting host feed efficiency (Ortigues and Doreau, 1995) and possibly modifying the intestinal microbiota composition. Although certain evidence from other livestock species (e.g., sheep) exists (Rodríguez et al., 2003), the impact of the feed intake level on the structure of the intestinal microbiota community has not been elucidated in chickens of diverging FE. In order to design nutritional strategies to effectively improve chicken's FE, the relationship among chicken's feed intake, intestinal microbiota, and host nutritional metabolism needs to be clarified to better understand the underlying modes of action for the divergence in FE. Especially, it will be helpful to clarify whether the differences in the feed intake between high and low feed-efficient chickens are the only driving force behind shifts in taxa abundances or whether other FE-associated differences in host physiology are major factors for the FE-associated variation in the bacterial community composition. To investigate the effect of the feed intake level on the FE-associated variation in the chicken intestinal microbiota, all chickens should eat the same amount of feed, irrespective of chicken's FE, which can be achieved by feeding chickens restrictively. Feed restriction in chicken rearing is generally used to prevent metabolic disorders (e.g., sudden death syndrome and ascites) and has been shown to manipulate carcass composition and FE in chickens (Van der Klein et al., 2016). For this reason, quantitative or qualitative restriction of feed may be a possible dietary strategy to improve chicken's FE.

Drastic alterations in the intestinal microbiota occur naturally in chickens from birth to market weight (Oakley et al., 2014). Any dietary interventions applied to improve chicken's FE will therefore modify the successional changes in the intestinal microbiota, which may modify the microbe-host interactions. To study the intestinal microbiota of chickens at multiple time points, it may be reasonable to collect fecal samples from the same animal in order to reduce the bias from inter-animal variation in the microbiota. Although not being completely representative for the intestinal microbiota, the fecal microbiota of chickens were shown to be qualitatively similar but quantitatively different to the cecal microbiota (Stanley et al., 2015). In order to investigate successional changes to dietary strategies, fecal samples may be therefore effectively used to detect some shifts and responses of the intestinal microbiota (Stanley et al., 2015).

Our objective was to investigate the effect of restrictive vs. *ad libitum* feeding on the fecal microbiota at 16 and 29 days posthatch (dph) as well as on the FE and nutrient retention at 4 weeks of age in low and highly feed-efficient chickens. In order to investigate the relationships between the feed intake level and the microbiota in feces as well as to identify the most influential bacterial taxa for chicken's FE, performance and nutrient retention, we performed supervised sparse partial least-squares (sPLS) regression and relevance networking analysis. We hypothesized that, regardless of chicken's FE, a quantitative restriction of the feed may improve the intestinal nutrient retention and modulate the abundance of the predominant bacterial taxa in chickens, whereby the effect of restrictive feeding may be stronger with increasing age of the chicken due to the continuously increasing feed intake. We also hypothesized that FE-related differences in the fecal microbiome should diminish when low and high feed-efficient chickens are fed the same amount of feed. In the present study, we used the residual feed intake (RFI) as metric for FE, with low RFI (negative) values representing high FE, and high RFI (positive) values representing low FE. Chicken's individual RFI value was determined for the whole experimental period (9–30 dph) at the end of the trial.

## Materials and methods

### Ethical statement

All experimental procedures including animal handling and treatment were approved by the institutional ethics committee of the University of Veterinary Medicine Vienna and the Austrian national authority according to paragraph 26 of Law for Animal Experiments, Tierversuchsgesetz 2012—TVG 2012 (GZ 68.205/0131-II/3b/2013).

### Animals, housing, and experimental design

A total of 112 one-day-old Cobb 500 female and male broiler chicks were obtained from a commercial hatchery (Brüterei Schlierbach GmbH, Pettenbach, Austria) and used in two consecutive replicate batches (56 chickens per batch). Two more females (*n* = 57) than males (*n* = 55) were used in the experiment, with one more female and one less male in batch 1 in comparison to batch 2. Housing and environmental conditions have been previously described (Metzler-Zebeli et al., 2016b). Upon arrival, 5 to 6 chicks of the same sex were group-housed in stainless steel metabolic cages until 8 dph. Afterwards, chickens were individually housed from 9 dph until the end of the experiment (32 dph) to determine the individual feed intake of each chicken. All chickens were fed the same starter (1–8 dph), grower (9–20 dph), and finisher (21–32 dph) corn-soybean meal based diets (Table S1) and had free access to demineralized water from manual drinkers. From 1 to 8 dph, all chicks had *ad libitum* access to feed to ensure sufficient feed intake in the first days of life. From 9 to 32 dph, chickens were randomly assigned to 2 different treatments. Half of the chickens had *ad libitum* access to feed (*n* = 29 females; and *n* = 28 males), whereas the other half were restrictively fed (*n* = 28 females; and *n* = 27 males). Separately per sex, the daily feed allocation of restrictively fed chickens was aimed to correspond to 90–95% of the average *ad libitum* feed intake observed in our previous chicken trial where we determined chicken's individual feed intake daily (Metzler-Zebeli et al., 2016b). Using these data, the expected feed intake of an “average chicken” was estimated for every single experimental day. Because differences between the previous and current experiment were to be expected and to ensure restrictive feeding of all chickens in this group, the amount of provided feed was additionally adjusted daily toward the female and male chicken with the lowest feed intake using the feed intake data from the day before. Our aim was to have empty feeders in the restrictively fed chicken group the next morning. For both treatment groups feed was provided at 9:00 a.m., and feeders were refilled at 3:00 p.m. Diets were free of antibiotics and coccidiostatics.

### Sample collection

Exreta samples were collected from all chickens in the two replicate batches. Excreta collection was facilitated by putting a tray, covered with parchment paper, under each cage. Feces collection for the fecal microbiota occurred on 16 and 29 dph. Because the intestinal origin of the chicken feces determines the fecal bacterial composition (Sekelja et al., 2012); only freshly dropped fecal samples of a paste-like texture and light brown color without the uric acid-containing white were aseptically collected within 10 min after defecation, snap frozen in liquid N_2_, and stored at −80°C. On 28 dph, fresh excreta samples were collected and stored at −20°C until analysis for NH_3_ concentration and pH. Excreta samples for analysis of DM content and retention of nutrients (DM, crude ash, CP, and P) were collected on 31 and 32 dph and stored at −20°C.

### Determination of feed efficiency

Feed refusals of each chicken were collected daily before morning feeding, and feed spills were collected weekly before recording individual feed intake on 9, 14, 21, 28, and 30 dph. Chickens were weighed upon arrival and on 7, 9, 14, 21, 28, and 30 dph. Chickens were ranked on their FE based on RFI. The RFI was determined for the period from 9 to 30 dph. For this, TFI, metabolic mid-test BW (MMW), and TBWG were calculated between 9 and 30 dph. A nonlinear mixed model (SAS Stat Inc., version 9.4; Cary, NC, USA) was used to estimate chicken's RFI as the residuals over the test period according to the following equations (Metzler-Zebeli et al., 2016b). The MMW was calculated as MMW = {[BW at 9 dph (g) + BW at 30 dph, (g)]/2}^0.75^. The RFI was calculated as RFI (g) = TFI – (a_1_ + b_1_ × MMW + b_2_ × TBWG), in which a_1_ is the intercept and b_1_ and b_2_ are partial regression coefficients of MMW and TBWG on TFI, respectively. Based on this calculation, high feed-efficient chickens were represented by low (negative) RFI values, whereas low feed-efficient chickens had high (positive) RFI values. Regression analysis was performed individually for chickens in each of the two batches. In each replicate batch, separately for females and males and balanced for batch, the chickens with the lowest RFI and highest RFI values were selected in the *ad libitum* and restrictively fed chicken groups. For both batches together, a total of 14 low RFI (*n* = 7 per sex) and 15 high RFI (*n* = 8 females; and *n* = 7 males) *ad libitum* fed chickens, and 14 low RFI (*n* = 7 per sex) and 14 high RFI (*n* = 7 per sex) restrictively fed chickens were selected as the extremes in RFI and were retrospectively chosen for analysis of excreta characteristics, nutrient retention, and fecal microbiota composition.

### Analytical methods

#### DNA extraction, 16s rRNA sequencing and bioinformatic analysis

Total DNA was extracted from 250 mg of fecal samples (*n* = 114) using the PowerSoil DNA isolation kit (MoBio Laboratories Inc., Carlsbad, CA, USA) with some modifications as described previously (Metzler-Zebeli et al., 2016a). The DNA concentration was quantified using the Qubit 2.0 Fluorometer (Life Technologies, Carlsbad, CA, USA) with the Qubit dsDNA HS Assay Kit (Life Technologies).

An aliquot of each of the extracted DNA samples was sent to a commercial provider (Microsynth AG, Balgach, Switzerland) for 16S rRNA gene PCR amplification, library preparation, and Illumina MiSeq sequencing. The V3-V5 hypervariable region of the 16S rRNA gene was amplified using the primers 357F-HMP (5′-CCTACGGGAGGCAGCAG-3′) and 926R-HMP (5′-CCGTCAATTCMTTTRAGT-3′) to generate an approximate amplicon size of 570 bp (Peterson et al., 2009). Amplification of the 16S rRNA gene was performed using the KAPA HiFi HotStart PCR Kit (Roche, Baden, Switzerland), which included a high-fidelity DNA polymerase, according to the manufacturer's instructions. Libraries were constructed by ligating sequencing adapters and indices onto purified PCR products using the Nextera XT sample preparation kit (Illumina Inc., San Diego, CA, USA) according to the recommendations of the manufacturer. Equimolar amounts for each library were pooled and sequenced on an Illumina MiSeq Personal Sequencer (v3; Illumina Inc.) using a 300 bp read length paired-end protocol. All sample libraries were sequenced in the same sequencing run. After sequencing, FASTQ files were de-multiplexed, trimmed of Illumina adaptor residuals using cutadapt (version 1.8.1; https://cutadapt.readthedocs.org/) and the overlapping paired-end reads were stitched using Fast Length Adjustment of SHort reads (FLASH, version 1.2.11; http://ccb.jhu.edu/software/FLASH/) by Microsynth (Siegerstetter et al., 2017). Raw sequencing data are available in NCBI's BioProject SRA database under accession no. PRJNA430313.

Sequence data were analyzed with the Quantitative Insights Into Microbial Ecology (**QIIME**) package version 1.9.1 (Caporaso et al., 2010). After quality trimming of the stitched reads using a quality threshold of *q* < 15, the UCHIME method using the 64-bit version of USEARCH (Edgar, 2010; Edgar et al., 2011) and the GOLD database (drive5.com) were used to screen for and exclude chimeric sequences. Open-reference operational taxonomic unit (**OTU**) picking was done at 97% similarity level using UCLUST (Edgar, 2010) and the Greengenes database as a reference template (version 13_8) (http://qiime.org/home_static/dataFiles.html; DeSantis et al., 2006). Representative sequences of the most abundant OTUs that were differently affected by time point, restrictive feeding, and RFI were additionally classified against the National Center for Biotechnology Information (**NCBI**) nucleotide database using Blastn for taxonomic classification (https://blast.ncbi.nlm.nih.gov/). Rare OTUs with <10 sequences were removed. For α-diversity analyses (Shannon, Simpson, and Chao1), samples were rarefied to a depth of 10,000 sequences, which excluded two samples with fewer reads (female, low RFI, restrictive, 16 dph; and female, high RFI, *ad libitum*, 29 dph). For β-diversity analysis, statistical assessment of dissimilarity matrices (Bray-Curtis) derived from OTU data was performed with PERMANOVA using the “adonis2” function and illustrated in two-dimensional nonmetric multidimensional scaling (NMDS) ordination plots obtained with the “metaMDS” function in the vegan R package (version 2.5.2) (Oksanen et al., 2018).

### Chemical analyses

The NH_3_ concentration in excreta was determined using the indophenol method (Weatherburn, 1967). Light absorbance was measured at 655 nm using a photometer (UV-1800, Shimadzu Corporation, Kyoto, Japan). The dry matter content of excreta was determined by oven-drying (Memmert Model 500, Memmert GmbH, Schwabach, Germany) at 105°C overnight (Naumann and Basler, 2012). Excreta pH was measured in a 1:9 (vol/vol) dilution using a pH meter (Seven Multi TM, Mettler-Toledo GmbH, Schwerzenbach, Switzerland) equipped with an electrode (InLab 413 SG, Mettler-Toledo GmbH). Prior to the proximate nutrient analysis (Naumann and Basler, 2012), total excreta samples were first pooled per chicken, freeze-dried (Gamma 2–20, Martin Christ Gefriertrocknungsanlagen GmbH, Osterode am Harz, Germany), and ground through a 0.5 mm screen (Ultra Centrifugal Mill ZM 200, Retsch GmbH, Haan, Germany; Metzler-Zebeli et al., 2016b). Acid insoluble ash was analyzed in feed and feces (Naumann and Basler, 2012) and was used as inert marker for calculation of nutrient retention (Metzler-Zebeli et al., 2018).

### Statistical analyses

Feed efficiency, performance traits, excreta characteristics, nutrient retention, and fecal microbiota data were analyzed for normality using Shapiro-Wilk test with the UNIVARIATE procedure in SAS (SAS Stat Inc., version 9.4; Cary, NC, USA). Thereafter, data were subjected to ANOVA using the MIXED procedure in SAS. The fixed effects of batch, sex, restrictive feeding, RFI, and the two-way-interaction restrictive feeding × RFI were considered in the main model to analyze FE, performance traits, excreta, and nutrient retention data. Batch was considered as random effect in the final model. The individual chicken was the experimental unit. Sex was significant for the FE and performance data; therefore, a second model for these parameters was adjusted and data were additionally separately analyzed for females and males. For the microbiota data, the fixed effects of time point of feces collection and the 3-way-interaction time point × restrictive feeding × RFI were additionally included in the model. Fecal microbiota data of the same chicken at different time points were considered as repeated measures in the model. Chicken nested within batch was the experimental unit. Degrees of freedom were approximated using the Kenward-Roger method. Least squares means were computed using the pdiff statement. Differences were considered significant if *P* ≤ 0.05 and as trend if 0.05 < *P* ≤ 0.10. Only those bacterial families, genera, and OTUs, which comprised a relative abundance > 0.05% across both sexes and sampling time points were statistically analyzed.

To identify the most influential OTUs affecting chicken's phenotype, sPLS regression and relevance network analyses were performed using the “mixOmics” package (version 6.3) in R studio (Rohart et al., 2017) to integrate data of relative bacterial abundances at 16 and 29 dph with results for RFI, performance traits (TFI and TBWG between 9 and 14 dph and between 9 and 30 dph, respectively), and nutrient retention. Relevance network graphs from sPLS were obtained via the function network.

## Results

### Feed efficiency and performance traits

Chickens with extremely low and high RFI values were retrospectively selected in the restrictively and *ad libitum* fed chicken groups to compare their fecal microbiota and nutrient retention. Specifically, 14 low RFI and 15 high RFI *ad libitum* fed chickens, and 14 low RFI and 14 high RFI restrictively fed chickens were selected from the 112 chickens on trial. Chicken's individual total feed intake (TFI), total body weight gain (TBWG), and RFI values were determined for the experimental period between 9 and 30 dph. The RFI rank related differences in feed intake were detectable in *ad libitum* fed females, with low RFI females having a 432 g lower TFI than high RFI females as indicated by the restrictive feeding × RFI interaction (*P* = 0.010; Table 1), but not in *ad libitum* fed males. The feed amount provided to the restrictively fed chickens was calculated to represent a feed restriction of about 90–95% of *ad libitum* feeding using the feed intake data from a previous trial using the same chicken genetic and dietary composition (Metzler-Zebeli et al., 2017, 2018; Siegerstetter et al., 2017). Across both sexes, restrictively fed chickens ate 338 g less between 9 and 30 dph than *ad libitum* fed chickens (*P* < 0.001). Because low RFI chickens commonly eat less than high RFI chickens (Metzler-Zebeli et al., 2017), the feed restriction was less severe in the low RFI chickens (92% of *ad libitum* group) compared to the high RFI chickens (80% of *ad libitum* group). Furthermore, across both sexes, restrictively fed chickens gained 231.5 g less weight between 9 and 30 dph than their *ad libitum* fed counterparts (*P* < 0.001). In both sexes, low RFI and high RFI chickens clearly had distinct RFI values (*P* < 0.001). In females, the restrictive feeding × RFI interaction (*P* = 0.021) indicated that in high RFI females the RFI values were 153 g lower with restrictive compared to *ad libitum* feeding, whereas the RFI values were similar in low RFI females between the restrictive and *ad libitum* feeding groups. This resulted in RFI values which were 327 g lower with low RFI compared to high RFI in *ad libitum* fed females, whereas in restrictively fed chickens, this difference decreased to 152 g between low RFI and high RFI groups. In males, this effect was less pronounced. However, when comparing the least-squares means of the high RFI ranks for both the *ad libitum* and restrictively fed chicken groups, a similar trend (*P* < 0.10) for an improved RFI value for the restrictively fed males could be detected.

**Table 1:** Total feed intake (TFI), total BW gain (TBWG), and residual feed intake (RFI) values of low and high RFI broiler chickens fed either *ad libitum* or restrictively^1^.

	***ad libitum*** **feeding**		**Restrictive feeding**			***P-*****value**		
**Item**	**Low RFI**	**High RFI**	**Low RFI**	**High RFI**	**SEM**	**Restr.^2^**	**RFI**	**Restr. × RFI**
**BOTH SEXES**								
TFI, g	2337^b^	2620^a^	2110^c^	2171^c^	52.5	**<0.001**	**0.002**	**0.040**
TBWG, g	1696	1684	1501	1416	41.1	**<0.001**	0.242	0.376
RFI, g	−81^c^	226^a^	−67^c^	111^b^	23.0	**0.033**	**<0.001**	**0.007**
**FEMALES**								
TFI, g	2146^b^	2578^a^	1965^c^	2074^bc^	57.7	**<0.001**	**<0.001**	**0.010**
TBWG, g	1529	1610	1354	1328	47.9	**<0.001**	0.570	0.279
RFI, g	−56^c^	271^a^	−34^c^	118^b^	35.7	0.077	**<0.001**	**0.021**
**MALES**								
TFI, g	2529	2652	2255	2269	84.9	**0.001**	0.428	0.525
TBWG, g	1862	1753	1649	1503	65.9	**0.002**	0.066	0.784
RFI, g	−107	179	−99	104	29.0	0.256	**<0.001**	0.171

1 *Data are presented as least-square means ± pooled standard error of the mean (SEM). n = 7 per feeding level, RFI rank, and sex; except for n = 8 high RFI ad libitum females. TFI, TBWG, and RFI were calculated for the experimental period from 9 to 30 d post-hatch. Sex affected TFI, TBWG (P ≤ 0.001), and RFI (P ≤ 0.05)*.

2 *Restr., restrictive feeding*.

a, b, c *Different superscripts within a row indicate significant difference (P ≤ 0.05)*.

*The bold values should be self-explanatory. That are the P-value which indicate significance*.

### 16S rRNA sequencing

Fecal samples from 57 chickens from 16 to 29 dph each were analyzed using in-depth sequencing to assess the microbiota composition of *ad libitum* and restrictively fed chickens divergent in RFI. After quality and chimera filtering, a total of 2,558,238 reads with a mean of 22,441 (SD ± 5,465) sequences per sample and a mean read length of 550 ± 8 bp remained.

### Fecal microbiota composition and age-related microbiota shifts

**Figure 1:**
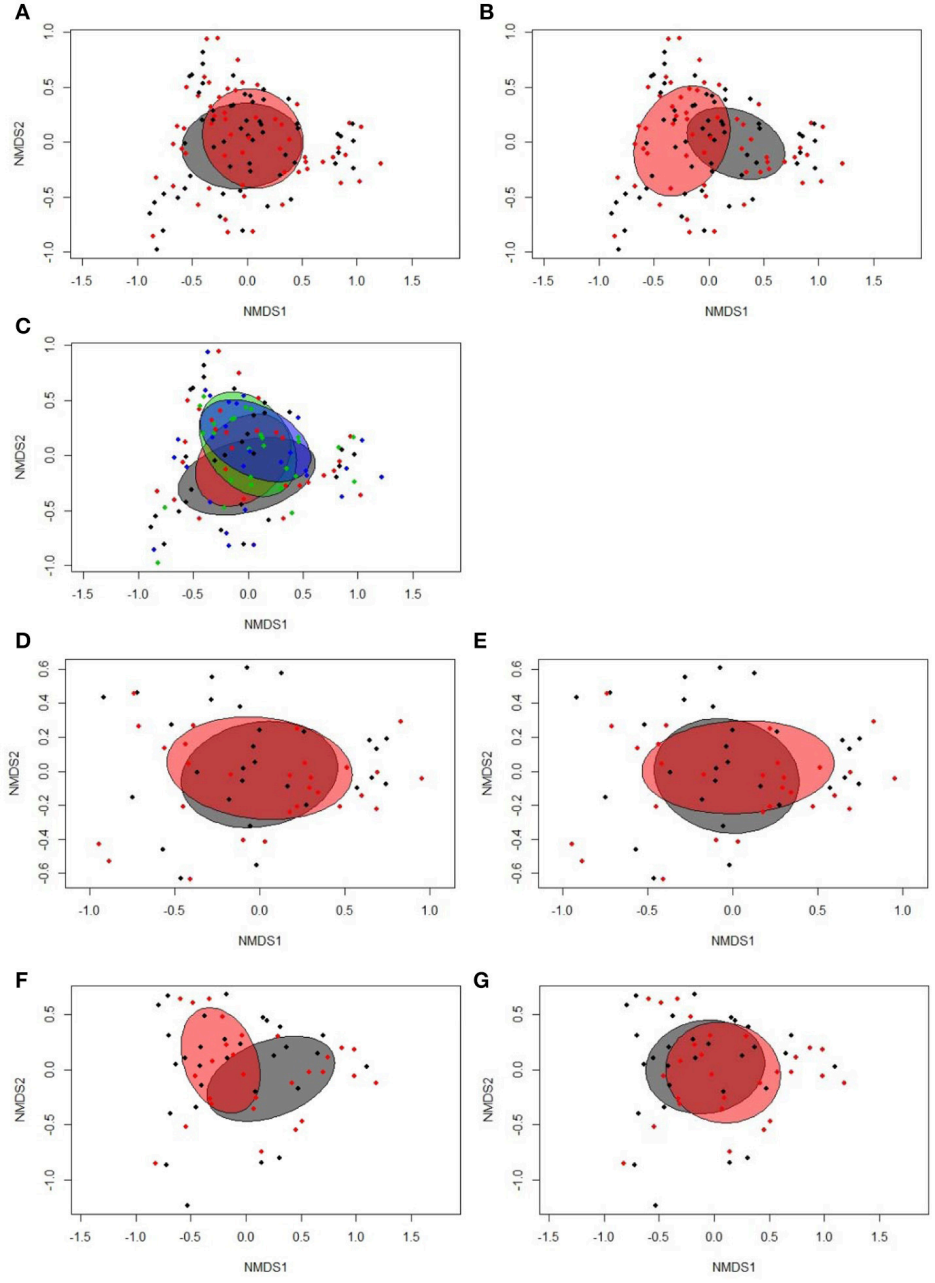
Two-dimensional non-parametric multidimensional scaling (NMDS) ordination plots of **(A)** fecal samples of females (gray) and males (red; Bray-Curtis dissimilarity, *P* = 0.398); **(B)** fecal samples at 16 (gray) and 29 days (red) post-hatch (dph; Bray-Curtis dissimilarity, *P* = 0.001); **(C)** fecal samples separated by treatment groups across time points (blue, restrictively fed low RFI; green, restrictively fed high RFI; red, *ad libitum* low RFI; gray, *ad libitum* fed high RFI; Bray-Curtis dissimilarity, *P* = 0.153); **(D)** fecal samples from *ad libitum* (gray) and restrictively (red) fed chickens on 16 dph (Bray-Curtis dissimilarity, *P* = 0.841); **(E)** fecal samples from low (gray) and high RFI (red) chickens on 16 dph (Bray-Curtis dissimilarity, *P* = 0.260); **(F)** fecal samples from *ad libitum* (gray) and restrictively (red) fed chickens on 29 dph (Bray-Curtis dissimilarity, *P* = 0.001); **(G)** fecal samples from low (gray) and high RFI (red) chickens on 29 dph (Bray-Curtis dissimilarity, *P* = 0.283). Low RFI *ad libitum, n* = 7 per sex and time point; high RFI *ad libitum, n* = 8 females at 16 dph and *n* = 7 females at 29 dph, and *n* = 7 males per time point; low RFI restrictive, *n* = 6 females at 16 dph and *n* = 7 females at 29 dph, and *n* = 7 males per time point; high RFI restrictive, *n* = 7 per sex and time point.

**Figure 2:**
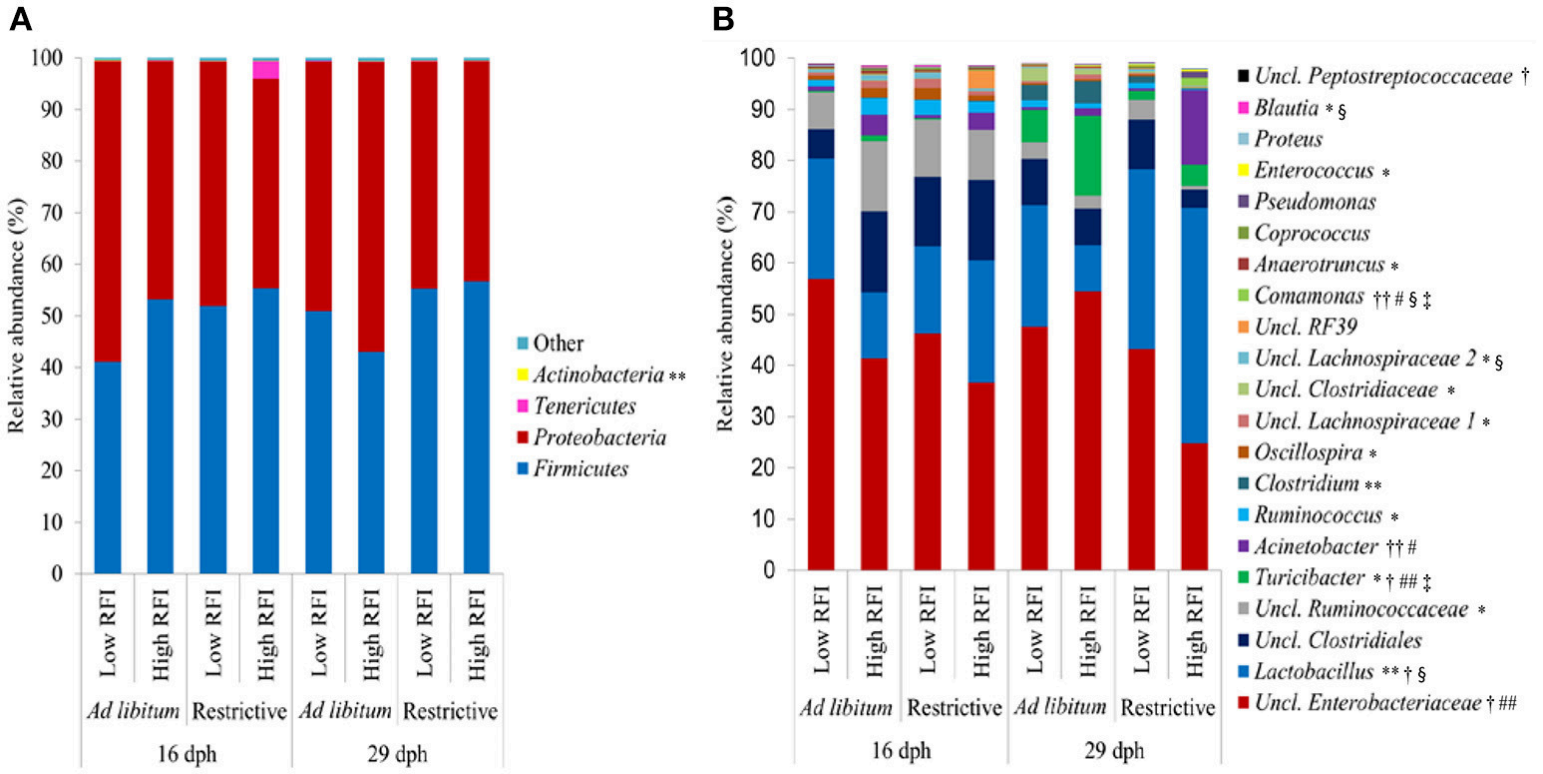
Relative abundance of **(A)** bacterial phyla and **(B)** most abundant bacterial genera (relative abundance > 0.5%) in feces at 16 and 29 days post-hatch (dph) in low and high residual feed intake (RFI) broiler chickens fed either *ad libitum* or restrictively. *P ≤ 0.05, effect for time point; **P ≤ 0.10, trend for time point effect; ^†^*P* ≤ 0.05, effect for restrictive feeding; ^††^*P* ≤ 0.10, trend for restrictive feeding effect; ^#^*P* ≤ 0.05, effect for RFI; ##*P* ≤ 0.10, trend for RFI effect; ^§^*P* ≤ 0.05 effect for restrictive feeding × RFI interaction; *P* ≤ 0.05 effect for time point × restrictive feeding × RFI interaction. Low RFI *ad libitum, n* = 7 per sex and time point; high RFI *ad libitum, n* = 8 females per time point, and *n* = 7 males per time point; low RFI restrictive, *n* = 7 per sex and time point; high RFI restrictive, *n* = 7 per sex and time point. Uncl., unclassified.

According to the Bray-Curtis dissimilarity, females and males had a similar overall bacterial community structure in feces (*P* = 0.398; Figure 1A). *Firmicutes* (50.9%) and *Proteobacteria* (47.9%) were the dominating phyla in chicken's feces across all chicken groups and time points (Figure 2A). At the genus level, an unclassified *Enterobacteriaceae* genus predominated (43.9%), followed by *Lactobacillus* (23.9%), an unclassified *Clostridiales* genus (10.0%), an unclassified *Ruminococcaceae* genus (6.5%), *Turicibacter* (3.7%), *Acinetobacter* (3.2%), *Ruminococcus* (1.7%), and *Clostridium* (1.1%) (Figure 2B). All other genera were of lower abundance (< 1.0%).

Bray-Curtis derived dissimilarity matrices showed a significant separation (*P* = 0.001) in chicken's bacterial community structure between 16 and 29 dph (Figure 1B), whereas α-diversity indices were similar across time points (*P* > 0.10; Table S2). Although few differences in the relative bacterial abundances between 16 dph and 29 dph at the phylum level were detected (Figure 2A), several families and within these families several genera and OTUs differed in their relative abundances between 16 dph and 29 dph (Table 2, Table S3, and Figure 2B). High-abundance *Ruminococcaceae* at the family level as well as an unclassified *Ruminococcaceae* (*P* < 0.001) and *Ruminococcus* (*P* = 0.002) at the genus level were 3.9-, 4.1-, and 2.7-fold more abundant at 16 dph than at 29 dph, respectively. In contrast, the high-abundance family *Turicibacteraceae* including the genus *Turicibacter* and *Turicibacter* OTU3 were more abundant (*P* < 0.001) at 29 dph than at 16 dph, however, only in *ad libitum* (15.5-, 15.5-, and 15.4-fold, respectively) and not in restrictively fed chickens as indicated by the time point × restrictive feeding × RFI interactions (*P* < 0.05).

**Table 2:** Differences in relative abundance (%) of most abundant bacterial families present in feces at 16 and 29 d post-hatch (dph) in low and high residual feed intake (RFI) broiler chickens fed either *ad libitum* or restrictively^1^.

	**16 dph**				**29 dph**									
	***ad libitum*** **feeding**		**Restrictive feeding**		***ad libitum*** **feeding**		**Restrictive feeding**			***P*****-value**				
**Item**	**Low RFI**	**High RFI**	**Low RFI**	**High RFI**	**Low RFI**	**High RFI**	**Low RFI**	**High RFI**	**SEM**	**TP^2^**	**FL^3^**	**RFI**	**FL × RFI**	**TP × FL × RFI**
*Enterobacteriaceae*	57.06	41.74	46.43	36.71	47.70	54.55	43.44	25.22	7.490	0.628	**0.015**	0.070	0.326	0.413
*Lactobacillaceae*	23.56	12.78	17.05	23.87	23.74	9.07	35.07	46.01	7.044	0.059	**0.014**	0.713	**0.043**	0.152
*Ruminococcaceae*	9.52	19.20	16.71	13.31	5.08	3.78	5.34	1.04	3.311	<**0.001**	0.902	0.944	0.092	0.410
*Turicibacteraceae*	0.25^b^^B^	1.18^b^	0.25^b^^B^	0.04^b^^B^	6.39^b^^A^	15.78^a^	1.65^b^	4.17^b^	2.417	<**0.001**	**0.016**	0.078	0.260	**0.038**
*Moraxellaceae*	0.91	4.09	0.66	3.42	0.51	1.50	0.51	14.54	2.678	0.327	0.092	**0.004**	0.079	0.084
*Lachnospiraceae*	1.53	3.26	4.00	1.74	0.91	1.23	1.16	0.25	0.744	**0.001**	0.920	0.612	**0.021**	0.480
*Clostridiaceae*	0.06	0.22	0.13	0.23	5.51	5.42	1.71	0.47	1.759	**0.016**	0.086	0.831	0.809	0.361
*Comamonadaceae*	0.15	0.14	0.05	0.27	0.01	0.03	0.05	1.45	0.266	0.234	**0.046**	**0.029**	**0.030**	0.061
*Enterococcaceae*	0.11	0.13	0.10	0.04	0.17	0.28	0.38	0.46	0.122	**0.011**	0.407	0.670	0.747	0.489
*Peptostreptococcaceae*	0.10	0.10	0.03	0.04	0.05	0.11	0.02	0.02	0.036	0.520	**0.017**	0.409	0.590	0.816

1 *Data are presented as least-square means ± pooled standard error of the mean (SEM). Low RFI ad libitum, n = 7 per sex and time point; high RFI ad libitum, n = 8 females per time point, and n = 7 males per time point; low RFI restrictive, n = 7 per sex and time point; high RFI restrictive, n = 7 per sex and time point. Sex affected Enterococcaceae (P ≤ 0.10)*.

2 *TP, time point*.

3 *Restr., restrictive feeding*.

a, b *Different superscripts within a row indicate significant difference (P ≤ 0.05)*.

A, B *Different superscripts within a row indicate a tendency (P ≤ 0.10)*.

*The bold values should be self-explanatory. That are the P-value which indicate significance*.

### Feeding level and feed efficiency-related microbiota shifts in feces

Bray-Curtis dissimilarity analysis indicated similar overall fecal bacterial communities across all chicken groups and time points (*P* = 0.153; Figure 1C). However, when analyzing the time points separately, significant separation in the fecal microbiota structure between the feeding level groups was detected at 29 dph (*P* = 0.001; Figure 1E) but not at 16 dph (*P* = 0.841; Figure 1D) and not for RFI groups at both time points (*P* > 0.10; Figures 1D–G). Moreover, α-diversity analysis showed that *ad libitum* fed chickens tended (*P* < 0.10) to have a less diverse bacterial community based on Shannon and Simpson indices compared to their restrictively fed counterparts. Furthermore, low RFI chickens tended (*P* = 0.062) to have a lower Simpson diversity compared to high RFI chickens (Table S2).

*ad libitum* fed chickens comprised more *Enterobacteriaceae* (*P* = 0.015; 1.3-fold), *Turicibacteraceae* (*P* = 0.016; 3.9-fold), and *Peptostreptococcaceae* (*P* = 0.017; 3.2-fold) at the family level, as well as at the genus level, an unclassified *Enterobacteriaceae* genus (*P* = 0.015; 1.3-fold), *Turicibacter* (*P* = 0.016; 3.9-fold), and an unclassified *Peptostreptococaceae* genus (*P* = 0.018; 3.2-fold) compared to restrictively fed chickens (Table 2, Figure 2B). The time point × restrictive feeding × RFI interactions (*P* = 0.038) for *Turicibacteraceae* and *Turicibacter*, however, indicated that only the 29-dph-old *ad libitum* fed high RFI chickens comprised 7.9-fold more of these bacteria compared to the other groups. In contrast, the family *Lactobacillaceae* and genus *Lactobacillus* were more abundant with restrictive compared to *ad libitum* feeding, but only in high RFI chickens (3.2-fold) as indicated by the restrictive feeding × RFI interactions (*P* = 0.043). Both restrictive feeding (*P* < 0.05) and high RFI rank (*P* < 0.05) were associated with increased abundances of the family *Comamonadaceae* and genus *Comamonas*. However, this effect was mainly due to their approximately 12-fold greater abundances in restrictively fed high RFI chickens compared to all other groups. In contrast, the family *Lachnospiraceae* and an unclassified *Lachnospiraceae* genus were oppositely affected by restrictive feeding and RFI rank but only at 16 dph as indicated by the restrictive feeding × RFI interactions (*P* < 0.05). Furthermore, the family *Moraxellaceae* and its genus *Acinetobacter* were 9.1-fold more abundant in high RFI compared to low RFI chickens (*P* = 0.004; and *P* = 0.005, respectively). In contrast, the restrictive feeding × RFI interactions (*P* = 0.043) for *Lactobacillaceae* and *Lactobacillus* indicated an increase in abundance (2.2-fold) in low RFI compared to high RFI chickens when *ad libitum* fed. Respective changes in OTU abundances and identity similarities of the most abundant and differently responding OTUs across treatment groups and time points to cultured organisms are presented in Tables S3, S4, respectively.

### Excreta characteristics and nutrient retention

The ammonia (NH_3_) concentration in excreta was about 24 μmol/g greater in high RFI than low RFI chickens (*P* = 0.017; Table 3). Furthermore, in high RFI chickens, the dry matter content of the excreta was 2.1% greater with *ad libitum* than restrictive feeding as indicated by the restrictive feeding × RFI interaction (*P* = 0.021). Restrictive feeding × RFI interactions (*P* < 0.05) indicated greater retention of dry matter, crude ash, crude protein, and phosphorus in restrictively fed high RFI chickens compared to all other groups.

**Table 3:** Excreta characteristics and retention of nutrients in low and high residual feed intake (RFI) broiler chickens fed either *ad libitum* or restrictively^1^.

**Item**	***ad libitum*** **feeding**		**Restrictive feeding**			***P-*****value**		
	**Low RFI**	**High RFI**	**Low RFI**	**High RFI**	**SEM**	**Restr.^2^**	**RFI**	**Restr. × RFI**
DM content, %	18.6^ab^^B^	20.1^a^^A^	19.3^ab^	18.0^b^	0.60	0.282	0.901	**0.021**
pH	6.8	6.8	6.7	6.6	0.14	0.344	0.976	0.703
Ammonia, μmol/g fresh sample	63.4	96.3	80.6	95.0	9.62	0.413	**0.017**	0.342
**RETENTION OF, %**								
DM	85.2^b^	85.1^b^	84.5^b^	88.4^a^	0.81	0.124	**0.022**	**0.015**
Crude ash	57.9^b^	57.7^b^	56.1^b^	66.1^a^	2.22	0.141	**0.033**	**0.026**
CP	81.6^b^	79.8^b^	80.7^b^	85.1^a^	1.13	0.051	0.250	**0.009**
Phosphorus	65.0^b^	62.5^b^	64.2^b^	73.1^a^	1.81	**0.010**	0.086	**0.003**

1 *Data are presented as least-square means ± pooled standard error of the mean (SEM). n = 7 per feeding level group, RFI rank, and sex; except for n = 8 high RFI ad libitum females*.

2 *Restr., restrictive feeding*.

a, b *Different superscripts within a row indicate significant difference (P ≤ 0.05)*.

A, B *Different superscripts within a row indicate a tendency (P ≤ 0.10)*.

*The bold values should be self-explanatory. That are the P-value which indicate significance*.

### Relevance networks

**Figure 3:**
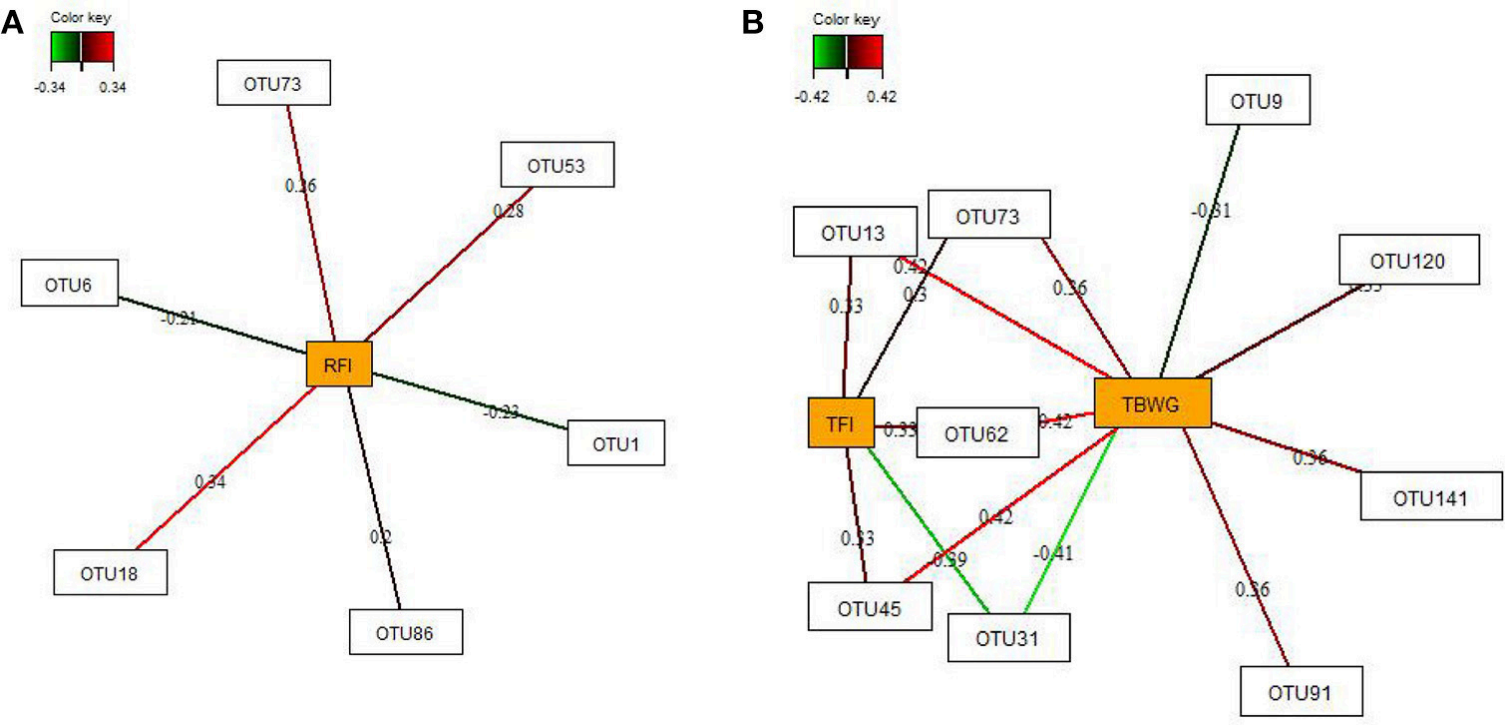
Determination of potential key operational taxonomic units (OTUs) at 16 days post-hatch for **(A)** residual feed intake (RFI) and **(B)** performance traits [total feed intake (TFI) and total body weight gain (TBWG) between 9 and 14 days post-hatch] in chickens ranked on RFI and fed either *ad libitum* or restrictively. Covariations between the relative abundances of bacterial OTUs in the feces and RFI, and performance traits using sparse partial least squares regression. The OTUs were included in the analysis if they occurred in at least half of the chickens. The network is displayed graphically as nodes (OTUs and performance traits) and edges (biological relationships between the nodes), with the edge color intensity indicating the level of the association: red = positive, and green = negative. Only the strongest pairwise associations were displayed, with score threshold depending of the respective association. The association scores are indicated under each edge. The cut-off threshold for the pairwise associations scores were 0.20 (positive and negative) in **(A)**, and 0.30 (positive and negative) in **(B)**.

**Figure 4:**
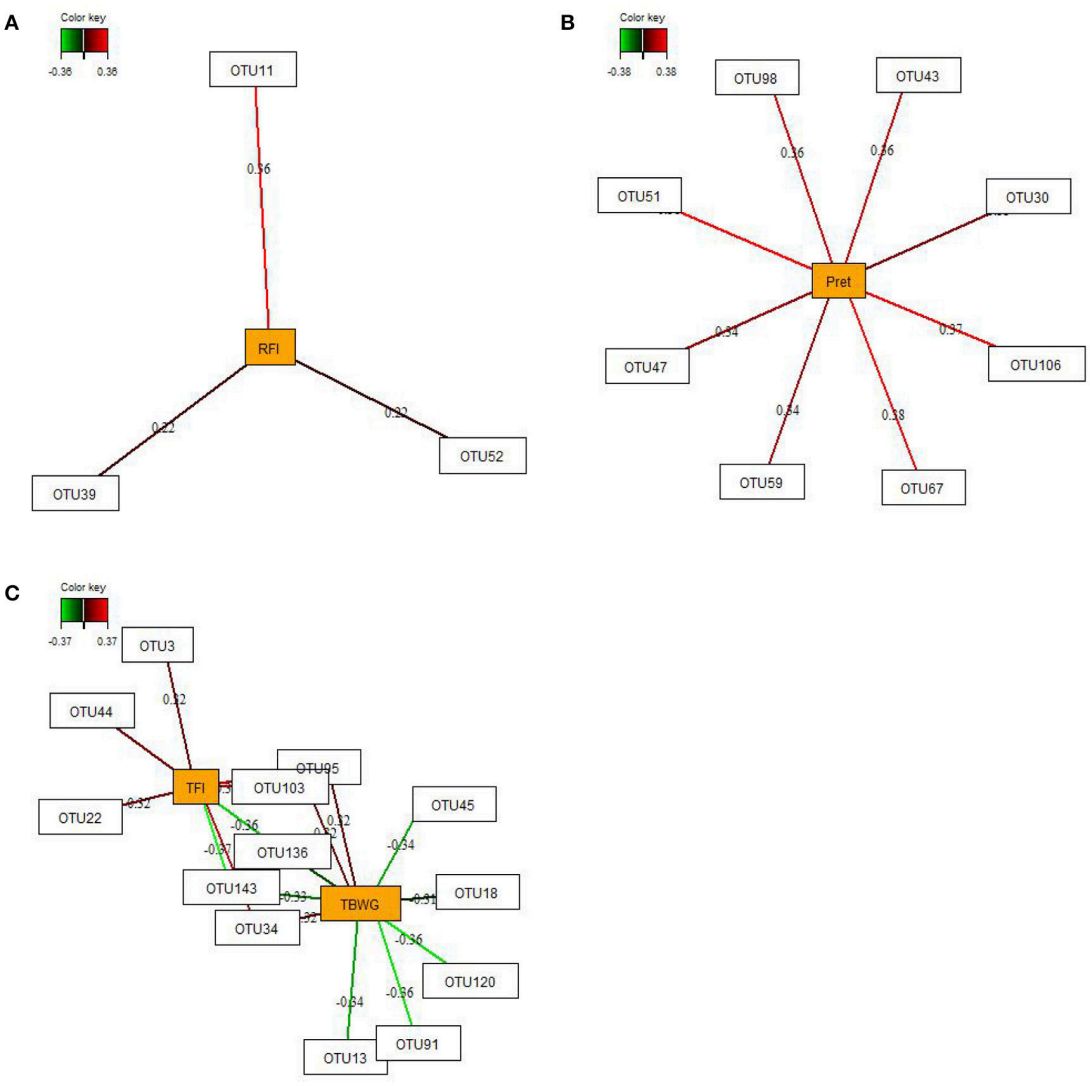
Determination of potential key operational taxonomic units (OTUs) at 29 days post-hatch for **(A)** residual feed intake (RFI), **(B)** phosphorus retention (Pret), and **(C)** performance traits (total feed intake (TFI) and total body weight gain (TBWG) between 9 and 30 days post-hatch) in chickens ranked on RFI and fed either *ad libitum* or restrictively. Covariations between the relative abundances of bacterial OTUs in the feces and RFI, Pret, and performance traits using sparse partial least squares regression. The OTUs were included in the analysis if they occurred in at least half of the chickens. The network is displayed graphically as nodes (OTUs and Pret, and performance traits) and edges (biological relationships between the nodes), with the edge color intensity indicating the level of the association: red = positive, and green = negative. Only the strongest pairwise associations were displayed, with score threshold depending of the respective association. The association scores are indicated under each edge. The cut-off threshold for the pairwise associations scores were 0.20 (positive and negative) in **(A)**, and 0.30 (positive and negative) in **(B,C)**.

By employing sparse partial-least squares (sPLS) regression and relevance network analysis, a predictive model constructed from the relative abundance of OTUs was used to discover influential bacterial taxa (OTUs) at 16 and 29 dph on chicken RFI and performance traits and nutrient retention (only OTUs from 29 dph). The strongest pairwise associations as the most influential are presented for each network (Figures 3, 4). The association score cut-off threshold was determined as 0.20 (positive and negative) for the relationship between OTUs and RFI and as 0.30 (positive and negative) for the relationships between OTUs and performance traits and nutrient retention. At 16 dph (Figure 3), relevance networks indicated that 2 *Escherichia* OTUs (OTU1 and OTU6) positively correlated with RFI, whereas 3 *Klebsiella* OTUs (OTU18, OTU53, and OTU73) and 1 *Acinetobacter* OTUs (OTU86) positively correlated with RFI. *Acenitobacter* OTUs (OTU13, OTU45, OTU62) and *Klebsiella* (OTU73) were positively associated with TFI between 9 and 14 dph, whereas 1 *Ruminococcus* OTU (OTU31) negatively associated with TFI and TBWG between 9 and 14 dph. Also, *Clostridium* OTU9 negatively correlated with TBWG between 9 and 14 dph, whereas 3 *Acinetobacter* OTUs (OTU62, OTU91 and OTU141), *Klebsiella* OTU73, and *Escherichia* OTU120 were positively correlated with TBWG between 9 and 14 dph. Moreover, relevance networks for OTUs in feces collected on 29 dph (Figure 4) showed that *Anaerobacterium* OTU11 and 2 *Clostridium* OTUs (OTU39 and OTU52) were positively correlated with RFI. Six OTUs were positively correlated with TFI between 9 and 30 dph, including 5 *Turicibacter* OTUs (OTU3, OTU22, OTU34, OTU44, and OTU103) and 1 *Escherichia*/*Shigella* OTU (OTU95), whereas 2 *Lactobacillus* OTUs (OTU136 and OTU143) were negatively associated with TFI between 9 and 30 dph. *Lactobacillus* OTU136 and OTU143 further negatively correlated with TBWG between 9 and 30 dph, as well as 3 *Acinetobacter* OTUs (OTU13, OTU45, and OTU91), *Klebsiella* OTU18, and *Escherichia*/*Shigella* OTU120. In contrast, increased abundance of *Turicibacter* OTU34, OTU95, and OTU103 was positively associated with TBWG between 9 and 30 dph. Moreover, 8 *Lactobacillus* OTUs were positively correlated with phosphorus retention.

## Discussion

In order to manipulate host FE, it is important to understand the role that the intestinal microbiota play for host FE. Characteristic differences in low and high RFI chickens are the FE-associated variation in feed intake and intestinal microbiota composition. Therefore, clarification was needed whether chicken's feed intake is the main factor behind FE-associated differences in the intestinal microbiota or whether host-related factors are behind these FE-related bacterial differences. This study provides valuable data for the cause and effect relationships between the feed intake level, the fecal microbiota composition and host physiology in low and high RFI chickens. By imposing the same feed intake on all chickens, the present results indicate that some FE-associated bacterial abundances (e.g., *Turicibacter*) in feces were caused mainly by chicken's feed intake, whereas *Enterobacteriaceae* and *Lactobacillus* appeared to be more affected by the host physiology-related nutrient retention. Concurrently, by decreasing the RFI value in the restrictively fed high RFI chickens, the present results showed that a similar feed intake would decrease the difference in chicken's actual RFI values between FE ranks, with the effect being stronger in females in the present study.

Due to qualitative but not quantitative similarities between the fecal and the microbiota in chicken's large intestine, it has been proposed that fecal samples may be used to identify responses of the intestinal microbiota to nutritional strategies (Stanley et al., 2015). Therefore, the observed differences in the fecal microbiota in the present study may reflect alterations in the microbiota of the distal intestine. Nevertheless, the present changes in the fecal microbial community are likely not applicable to predict feed restriction and RFI-associated effects on the microbiota in the upper digestive tract (Yan et al., 2017). Overall, we could identify age-, feed intake level- and RFI-associated bacterial profiles in feces of the present chicken population. In comparing the two time points, the fecal bacterial communities were more affected by the restrictive feeding at 29 dph than at 16 dph, demonstrating the importance of the greater feed intake with increasing age of the chicken for the bacterial community. Against our hypothesis, low and high RFI chickens of the restrictively fed chicken group did not have similar bacterial microbiota profiles in feces at 16 and 29 dph compared to the *ad libitum* fed chicken group as indicated by time point × restrictive feeding × RFI interactions and Bray-Curtis dissimilarity, emphasizing that other host-related factors also influenced the bacterial composition in feces. Moreover, we could only identify few bacterial taxa that showed a clear association with chicken's RFI rank for both time points. Our data greatly suggest that bacterial substrate availability was a major factor for the dynamics of the major bacterial populations in feces of *ad libitum* and restrictively fed low and high RFI chickens. Time point × restrictive feeding × RFI interactions may indicate that maturational changes in the intestinal microbiota and host physiology (e.g., mucin secretion and nutrient transporter expression; Gilbert et al., 2008; Schokker et al., 2015; Pender et al., 2017) modified the bacterial dependencies from chicken's feed intake on 16 and 29 dph. In fact, restrictively fed high RFI chickens comprised fewer nutrients in feces compared to the other 3 chicken groups at the end of the experimental period, which may be one probable explanation for the diverging bacterial profiles in restrictively fed chickens. In considering both the present feed intake and nutrient retention data, *ad libitum* fed high RFI chickens had the highest amount of nutrients and restrictively fed high RFI chickens the lowest amount of nutrients in the distal intestine and cloacae. The greater nutrient retention in restrictively fed high RFI chickens may have been a physiological adaptation to compensate for their lower feed intake in order to meet their greater energy and nutrient requirements compared to low RFI chickens (Bottje and Carstens, 2009), thereby allowing the chickens to maximize utilization of dietary energy and nutrients for growth, resulting in improved FE. Overall, the tendency for greater species richness and evenness in the restrictively compared to *ad libitum* fed chickens at both time points may support a prolonged intestinal retention time (Rodríguez et al., 2003), which would have increased the contact time between the intestinal microbiota and non-digested dietary material in digesta, allowing a broader variety of species to grow.

Feed efficiency-associated differences in the fecal abundances were, for instance, detectable for *Enterobacteriaceae*, being indicative for low RFI mainly at 16 dph, and *Moraxellaceae* being enriched in high RFI chickens across time points. However, when looking at the data in detail, the abundances of both families at 29 dph became enriched only in the restrictively fed high RFI chickens but not in their *ad libitum* fed counterparts. Overall, the sPLS regression and relevance network analysis supported those findings, identifying 2 *Escherichia*-OTU as the best discriminant bacteria for low RFI, and 3 *Klebsiella*- and 1 *Acinetobacter*-OTU as marker bacteria at 16 dph for high RFI in the present study. At 29 dph, in turn, 3 *Clostridium*-OTUs with an average abundance of 0.01–0.19% in chicken's feces at 29 dph were strongly associated with host RFI. However, due to the partly low sequence identity to reference strains, it can be only speculated whether these OTUs benefited from the greater substrate availability in host digesta of the distal intestine and/or the increased fermentative activities of other bacteria in high RFI chickens.

The nutrient gradients in digesta from the distal intestine and cloacae may have been reflected by the opposite results for the fecal abundances of the two predominant families *Enterobacteriaceae* and *Lactobacillaceae* at 29 dph. Accordingly, the decrease in proteolytic *Escherichia*/*Shigella* at 29 dph in feces was probably the consequence of the lower crude protein concentration in the restrictively fed high RFI chickens (Apajalahti and Vienola, 2016). The depletion of digesta with more easily fermentable substrate (e.g., protein and starch) may have promoted the growth of bacteria able to degrade complex carbohydrates and host mucin. Although both *Escherichia* and *Lactobacillus* have the metabolic capability to colonize and utilize intestinal mucins as a source of carbon, protein, and energy (Edens, 2003; Conway and Cohen, 2015), *Lactobacillus* may have had an additional colonization advantage against *Escherichia* through metabolic features, such as production of *Escherichia*-inhibiting antimicrobials and lactic acid (Cisek and Binek, 2008). Likewise, proteolytic activity and the ability to utilize amines, such as histamine, for growth may explain the enrichment with *Acinetobacter* species in restrictively fed high RFI chickens (Nemec et al., 2010).

Present sPLS regression and relevance network analysis further illustrated the importance of the feed intake level for the predominant bacteria (i.e., *Enterobacteriaceae, Turicibacter, Lactobacillus*, and *Acinetobacter*) in feces on 16 and 29 dph. However, dependencies of bacterial abundances from chicken's feed intake differed between 16 and 29 dph. While mainly *Acinetobacter*-OTUs benefited from the higher TFI on 16 dph, there were especially *Turicibacter*-OTUs on 29 dph that showed a strong dependency from chicken's feed intake. Although both *Lactobacillus* and *Turicibacter* species may utilize (resistant) starch (Gänzle and Follador, 2014; Sun et al., 2016), other dietary factors and microbe-microbe-interactions may explain that *Turicibacter* dominated this ecological niche, resulting in opposite abundance patterns across the 4 chicken groups. In line with this assumption, relevance network analysis indicated positive relationships between phosphorus retention and the fecal abundance of several *L*. *salivarius* and *L*. *crispatus* OTUs, suggesting increased bacterial growth with lower fecal phosphorus concentrations. The question arises as to whether this relationship might indicate a lower phosphorus requirement for growth in *Lactobacillus* species or whether other bacteria with greater phosphorus demands were replaced by *Lactobacillus* OTUs (Metzler and Mosenthin, 2008; Metzler-Zebeli et al., 2010). In line with the present findings, Ptak et al. (2015) found an increased ileal abundance of *Lactobacillus* spp./*Enterococcus* spp. associated with low levels of intestinal phosphorus and calcium.

Finally, it is worth mentioning that low RFI has been associated with improved dietary utilization of crude protein (Aggrey et al., 2014; Metzler-Zebeli et al., 2018), which was in contrast to the results for the *ad libitum* fed low and high RFI chickens, showing a similar retention of dry matter, crude ash, crude protein, and phosphorus in the present study. This might have been related to the fact that chickens in the present study were 5 to 7-days younger compared to previous studies (Aggrey et al., 2014; Metzler-Zebeli et al., 2018). Moreover, the lower TFI in restrictively fed low RFI chickens compared to their *ad libitum* fed low RFI counterparts was apparently not sufficiently different to increase the nutrient retention in the restrictively fed low RFI chickens.

In conclusion, the present study provided data for the contribution of the feed intake level in the FE-associated variation in the fecal microbiota in low and high RFI chickens. Our data demonstrated that bacterial substrate availability was an important factor for the dynamics of the dominant bacterial populations in feces of *ad libitum* and restrictively fed low and high RFI chickens. Overall, restrictive feeding affected the bacterial communities more at 29 dph than at 16 dph, indicating the importance of the greater feed intake with increasing age on chicken's fecal microbiota. However, feeding chickens the same amount of feed did not result in similar bacterial profiles between low and high RFI chickens at 16 and 29 dph, which may have been related to the improved nutrient retention in restrictively fed high RFI chickens. This not only altered the bacterial substrate availability but likely explains the decreased RFI value in restrictively fed high RFI chickens. Dependencies between chicken's feed intake and fecal microbiota composition were further supported by relevance network analysis, showing positive (*Escherichia*/*Shigella* and *Turicibacter*) and negative relationships (*Lactobacillus*) between TFI and bacterial abundances.

## Author contributions

BM-Z, EM, PL, and NO conceived and designed the experiments. S-CS and BM-Z performed the experiments and together with RP collected samples. S-CS, BM-Z, and RP analyzed the data. BM-Z wrote the statistical codes. S-CS and BM-Z interpreted the data. S-CS and BM-Z drafted the manuscript. RP, PL, QZ, EM, and NO revised the manuscript. BM-Z primary responsibility for the final content. All of the authors read and approved the final manuscript.

### Conflict of interest statement

The authors declare that the research was conducted in the absence of any commercial or financial relationships that could be construed as a potential conflict of interest.
