# Supplementary material for: Feed Restriction Modulates the Fecal Microbiota Composition, Nutrient Retention, and Feed Efficiency in Chickens Divergent in Residual Feed Intake
Source: Front Microbiol. 2018 Nov 19;9:2698. doi: 10.3389/fmicb.2018.02698 (PMC6254087; doi:10.3389/fmicb.2018.02698)
Supplement: Supplementary file 1 [file Data_Sheet_1.PDF]

Running Title: Feeding level and chicken's feed efficiency

Feed restriction modulates the fecal microbiota composition, nutrient retention and feed efficiency in chickens divergent in residual feed intake

Sina-Catherine Siegerstetter[1], Renée M. Petri[1], Elizabeth Magowan[2], Peadar G. Lawlor[3], Qendrim Zebeli[1], Niamh E. O´Connell[4], Barbara U. Metzler-Zebeli[1]

[1]Institute of Animal Nutrition and Functional Plant Compounds, Department for Farm Animals and Veterinary Public Health, University of Veterinary Medicine Vienna, Vienna, Austria.

[2]Agri-Food and Biosciences Institute, Agriculture Branch, Hillsborough, Northern Ireland, UK.

[3]Teagasc, Pig Development Department, Animal & Grassland Research & Innovation Centre, Moorepark, Ireland.

[4]Institute for Global Food Security, Queen´s University Belfast, Northern Ireland, UK.

***Correspondence:**
Dr. Barbara Metzler-Zebeli
barbara.metzler@vetmeduni.ac.at

Table S1. Dietary ingredients and chemical composition of diets (on as-fed basis)

Item	Starter<br>1 to 8 d post-hatch	Grower<br>9 to 20 d post-hatch	Finisher<br>21 to 30 d post-hatch
Ingredient (%)			
Corn	56.392	61.772	63.928
Soybean meal	37.032	31.432	29.068
Soybean oil	2.284	2.676	3.412
Monocalcium phosphate	1.460	1.412	1.240
Calcium carbonate	1.372	1.280	1.160
DL-Methionine	0.352	0.308	0.256
Natrium bicarbonate	0.204	0.212	0.136
Lysine-HCL 98	0.140	0.148	0.044
Salt	0.120	0.120	0.156
L-Threonine	0.044	0.040	0
Premix[1]	0.600	0.600	0.600
Analyzed chemical composition, g/kg DM			
DM	882	885	879
CP	228	207	194
Crude fat	51.1	47.5	61.0
Crude fiber	28.0	29.0	30.1
Crude ash	52.6	50.0	46.3
Starch	356	388	401
Sugar	55.0	49.9	45.4
Calcium	8.88	8.39	7.63
Phosphorus	6.90	7.05	6.66
ME, MJ/kg	12.0	12.0	12.4

[1]Provided per kilogram of complete starter diet (Garant - Tiernahrung GmbH, Pöchlarn, Austria): 12,500 IU of vitamin A, 5,000 IU of vitamin D_3, 75.0 mg of vitamin E, 6.0 mg of vitamin K3, 2.50 mg of vitamin B_1, 7.0 mg of vitamin B_2, 4.50 mg of vitamin B_6, 0.025 mg of vitamin B_{12}, 60.0 mg of nicotinic acid, 15.0 mg of pantothenic acid, 1.0 mg of folic acid, 0.25 mg of biotin, 1,582.065 mg of choline, 400.322 mg of choline chloride, 115.20 mg of betaine, 19.98 mg of ethoxyquin, 900.004 FTU of 6Phytase, 0.564 g of ß-glucan, 2.428 % of C 18:2, 2.804 % of polyenic acid, 30.059 mg of F-Xanto (total). Provided per kilogram of complete grower diet (Garant - Tiernahrung GmbH, Pöchlarn, Austria): 12,500 IU of vitamin A, 5,000 IU of vitamin D_3, 75.0 mg of vitamin E, 6.0 mg of vitamin K3, 2.50 mg of vitamin B_1, 7.0 mg of vitamin B_2, 4.50 mg of vitamin B_6, 0.025 mg of vitamin B_{12}, 60.0 mg of nicotinic acid, 15.0 mg of pantothenic acid, 1.0 mg of folic acid, 0.25 mg of biotin, 1,457.765 mg of choline, 400.322 mg of choline chloride, 115.20 mg of betaine, 19.98 mg of ethoxyquin, 900.004 FTU of 6Phytase, 0.618 g of ß-glucan, 2.692 % of C 18:2, 3.103% of polyenic acid, 30.866 mg of F-

Xanto (total). Provided per kilogram of complete finisher diet (Garant - Tiernahrung GmbH, Pöchlarn, Austria): 5,000 IU of vitamin D_3, 75.0 mg of vitamin E, 6.0 mg of vitamin K3, 2.50 mg of vitamin B_1, 7.0 mg of vitamin B_2, 4.5 mg of vitamin B_6, 0.025 mg of vitamin B_{12}, 60.0 mg of nicotinic acid, 15.0 mg of pantothenic acid, 1.0 mg of folic acid, 0.25 mg of biotin, 1,404.717 mg of choline, 400.322 mg of choline chloride, 115.20 mg of betaine, 19.98 mg of ethoxyquin, 900.004 FTU of 6Phytase, 0.639 g of ß-glucan, 3.098 % of C 18:2, 3.572 % of polyenic acid, 31.189 mg of F-Xanto (total).

Table S2. Differences in α-diversity indices in feces at 16 and 29 d post-hatch (dph) in low and high residual feed intake (RFI) broiler chickens fed either ad libitum or restrictively[1]

	16 dph				29 dph									
	Ad libitum feeding		Restrictive feeding		*Ad libitum* feeding		Restrictive feeding			*P* value				
Item	Low RFI	High RFI	Low RFI	High RFI	Low RFI	High RFI	Low RFI	High RFI	SEM	TP[2]	Restr.[3]	RFI	Restr.×RFI	TP×Restr.×RFI
Shannon	3.2	4.3	3.6	4.0	3.3	2.8	3.6	3.9	0.36	0.201	0.094	0.170	0.913	0.153
Simpson	0.65	0.77	0.70	0.75	0.67	0.64	0.71	0.80	0.049	0.724	0.052	0.062	0.658	0.373
Chao1	624	969	758	897	725	522	760	586	130.4	0.100	0.642	0.754	0.611	0.168

[1]Data are presented as least-square means ± pooled standard error of the mean (SEM). Low RFI *ad libitum*, *n* = 7 per sex and time point; high RFI *ad libitum*, *n* = 8 females at 16 dph and *n* = 7 females at 29 dph, and *n* = 7 males per time point; low RFI restrictive, *n* = 6 females at 16 dph and *n* = 7 females at 29 dph, and *n* = 7 males per time point; high RFI restrictive, *n* = 7 per sex and time point. Rarefaction depth of 10,000 sequences per sample removed 2 samples from the dataset (female, low RFI, restrictive, 16 dph; and female, high RFI, *ad libitum*, 29 dph).

[2]TP, time point.

[3]Restr., restrictive feeding.

Table S3. Differences in relative abundance (%) of most abundant operational taxonomic units (OTUs) in feces at 16 and 29 d post-hatch (dph) in low and high residual feed intake (RFI) broiler chickens fed either *ad libitum* or restrictively[1]

		16 dph				29 dph									
		Ad libitum feeding		Restrictive feeding		*Ad libitum* feeding		Restrictive feeding			*P* value				
Item	Taxonomy (Genus)[2]	Low RFI	High RFI	Low RFI	High RFI	Low RFI	High RFI	Low RFI	High RFI	SEM	TP[3]	Restr.[4]	RFI	Restr.×RFI	TP×Restr.×RFI
OTU1	*Escherichia*/*Shigella*	49.16	31.54	39.25	32.24	42.26	47.76	38.63	20.35	6.784	0.880	0.023	0.034	0.446	0.255
OTU2	*Lactobacillus*	9.70	4.80	7.73	12.34	12.27	5.85	12.71	20.66	3.707	0.105	0.058	0.908	0.030	0.763
OTU5	*Lactobacillus*	3.89	1.96	3.41	3.62	2.34	0.51	5.52	7.27	1.361	0.494	0.004	0.628	0.130	0.178
OTU4	*Agathobaculum*	3.95	7.03	5.60	4.46	1.01	0.76	1.35	0.29	1.192	<0.001	0.756	0.852	0.140	0.569
OTU3	*Turicibacter*	0.19^{bB}	0.90^{b}	0.20^{bB}	0.03^{bB}	4.76^{bA}	12.09^{a}	1.14^{b}	3.21^{b}	1.899	<0.001	0.019	0.077	0.270	0.042
OTU6	*Escherichia*/*Shigella*	3.47	2.29	2.92	2.26	3.33	3.79	3.10	1.71	0.503	0.527	0.030	0.037	0.306	0.265
OTU8	*Lactobacillus*	2.77	1.25	2.48	2.55	1.67	0.32	4.13	5.01	1.000	0.479	0.004	0.481	0.165	0.222
OTU9	[*Clostridium*]	1.35	5.21	3.22	3.58	0.78	1.53	1.30	0.17	1.452	0.019	0.889	0.372	0.213	0.661
OTU7	*Lactobacillus*	2.93	1.34	0.26	0.58	2.07	0.60	3.12	1.15	1.266	0.634	0.587	0.162	0.671	0.494
OTU10	[*Clostridium*]	0.97	2.68	2.68	1.59	0.70	0.54	1.75	0.55	0.881	0.094	0.488	0.759	0.118	0.769
OTU13	*Acinetobacter*	0.30^{b}	0.95^{b}	0.09^{b}	0.61^{b}	0.22^{b}	0.56^{b}	0.16^{b}	7.86^{a}	1.334	0.083	0.073	0.015	0.054	0.020
OTU18	*Klebsiella*	2.20	3.95	1.98	0.32	0.11	0.18	0.02	0.43	1.133	0.021	0.249	0.859	0.338	0.410
OTU15	*Agathobaculum*	1.04	1.91	1.57	1.14	0.30	0.41	0.36	0.07	0.339	<0.001	0.614	0.801	0.107	0.678
OTU21	*Lactobacillus*	0.71	0.31	0.60	0.69	0.48	0.10	1.18	1.43	0.265	0.265	0.002	0.546	0.127	0.170
OTU19	*Anaeromassilibacillus*	0.53	1.02	1.03	0.99	0.50	0.36	0.56	0.08	0.300	0.014	0.771	0.864	0.331	0.485
OTU20	*Lactobacillus*	0.16^{b}	0.81^{ab}	0.08^{b}	0.10^{b}	0.56^{ab}	0.15^{b}	1.31^{a}	1.19^{a}	0.362	0.054	0.328	0.898	0.732	0.048
OTU27	*Lactobacillus*	0.35	0.15	0.29	0.45	0.43	0.20	0.48	0.75	0.137	0.103	0.041	0.985	0.033	0.797
OTU22	*Turicibacter*	0.02^{bB}	0.09^{b}	0.02^{bB}	0.003^{bB}	0.61^{bA}	1.58^{a}	0.15^{b}	0.43^{b}	0.239	<0.001	0.018	0.069	0.267	0.029
OTU30	*Lactobacillus*	0.39	0.19	0.32	0.34	0.22	0.06	0.52	0.73	0.129	0.449	0.005	0.719	0.112	0.118
OTU39	[*Clostridium*]	0.22	0.63	0.87	0.20	0.13	0.53	0.08	0.01	0.245	0.040	0.678	0.931	0.060	0.229

OTU31	[*Ruminococcus*]	0.42	0.58	0.77	0.30	0.14	0.07	0.26	0.07	0.154	0.002	0.641	0.160	0.070	0.747
OTU45	*Acinetobacter*	0.10^{b}	0.27^{b}	0.02^{b}	0.19^{b}	0.05^{b}	0.18^{b}	0.05^{b}	1.62^{a}	0.280	0.115	0.097	0.010	0.063	0.032
OTU26	*Gracilibacter*	0.02	0.06	0.01	0.02	0.54	0.74	0.24	0.78	0.279	0.007	0.718	0.328	0.694	0.789
OTU16	*Anaerobacterium*	0.32	0.23	0.40	0.75	0.30	0.08	0.31	0.07	0.216	0.091	0.383	0.774	0.536	0.305
OTU43	*Lactobacillus*	0.18	0.08	0.11	0.37	0.34	0.06	0.43	0.84	0.125	0.008	0.005	0.458	0.007	0.204
OTU42	*Lactobacillus*	0.31	0.14	0.27	0.29	0.21	0.05	0.49	0.62	0.112	0.284	0.002	0.523	0.122	0.177
OTU35	*Eisenbergiella*	0.16	0.48	0.71	0.60	0.02	0.06	0.18	0.01	0.199	0.006	0.164	0.900	0.248	0.716
OTU47	*Lactobacillus*	0.19	0.08	0.12	0.30	0.32	0.05	0.39	0.68	0.112	0.017	0.012	0.761	0.013	0.265
OTU51	*Lactobacillus*	0.19	0.06	0.11	0.39	0.19	0.05	0.37	0.73	0.106	0.037	0.001	0.250	0.006	0.188
OTU52	[*Clostridium*]	0.24	0.55	0.77	0.18	0.06	0.17	0.02	0.01	0.181	0.002	0.957	0.733	0.078	0.264
OTU53	*Klebsiella*	0.22	0.94	0.23	0.12	0.03	0.04	0.02	0.19	0.208	0.039	0.254	0.179	0.260	0.117
OTU48	*Negativibacillus*	0.16	0.31	0.41	0.52	0.11	0.12	0.15	0.02	0.140	0.018	0.313	0.728	0.627	0.492
OTU33	*Escherichia/Shigella*	0.06^{c}	0.07^{bcB}	0.05^{c}	0.05^{c}	0.30^{bA}	0.69^{a}	0.19^{bc}	0.16^{bc}	0.083	<0.001	0.009	0.129	0.085	0.009
OTU60	[*Clostridium*]	0.11^{bc}	0.64^{a}	0.31^{b}	0.17^{bc}	0.09^{bc}	0.11^{bc}	0.05^{c}	0.005^{c}	0.100	<0.001	0.206	0.240	0.026	0.028
OTU32	*Saccharofermentans*	0.03	0.23	0.57	0.16	0.14	0.08	0.28	0.03	0.161	0.353	0.205	0.224	0.066	0.693
OTU59	*Lactobacillus*	0.10^{c}	0.08^{c}	0.06^{c}	0.17^{bc}	0.21^{bcC}	0.05^{cD}	0.30^{abBC}	0.50^{aA}	0.070	0.001	0.006	0.587	0.025	0.045
OTU67	*Lactobacillus*	0.13	0.06	0.09	0.25	0.14	0.03	0.26	0.51	0.076	0.050	0.001	0.312	0.010	0.150
OTU62	*Acinetobacter*	0.11	0.37	0.04	0.27	0.05	0.07	0.03	0.50	0.157	0.754	0.585	0.023	0.315	0.466
OTU56	*Anaerobacterium*	0.06	0.08	0.19	0.57	0.12	0.06	0.21	0.05	0.134	0.250	0.072	0.645	0.485	0.122
OTU82	*Klebsiella*	0.19	0.44	0.35	0.05	0.05	0.06	0.009	0.09	0.132	0.037	0.512	0.937	0.205	0.374
OTU86	*Acinetobacter*	0.10	0.17	0.05	0.77	0.008	0.08	0.02	0.05	0.182	0.084	0.303	0.084	0.232	0.217
OTU44	*Turicibacter*	0.01^{bB}	0.04^{b}	0.01^{bB}	0.003^{bB}	0.26^{bA}	0.64^{a}	0.06^{b}	0.17^{b}	0.097	<0.001	0.016	0.081	0.275	0.032
OTU68	*Flintibacter*	0.11	0.23	0.56	0.18	0.06	0.03	0.05	0.01	0.127	0.013	0.303	0.375	0.154	0.343
OTU77	*Oscillibacter*	0.19	0.38	0.28	0.16	0.02	0.09	0.05	0.002	0.091	0.001	0.494	0.719	0.122	0.883
OTU73	*Klebsiella*	0.13	0.45	0.15	0.09	0.04	0.05	0.03	0.19	0.095	0.076	0.446	0.122	0.421	0.083
OTU75	*Eisenbergiella*	0.04	0.21	0.37	0.44	0.04	0.04	0.02	0.006	0.116	0.005	0.132	0.475	0.733	0.250
OTU71	*Lactobacillus*	0.11	0.06	0.10	0.16	0.16	0.08	0.18	0.26	0.045	0.060	0.032	0.875	0.034	0.779

OTU76	*Lactobacillus*	0.07	0.07	0.10	0.11	0.13	0.12	0.18	0.16	0.039	0.023	0.161	0.880	0.986	0.947
OTU79	[*Clostridium*]	0.08	0.20	0.24	0.19	0.07	0.07	0.08	0.01	0.052	0.003	0.465	0.958	0.065	0.440
OTU74	*Agathobaculum*	0.13	0.26	0.21	0.16	0.04	0.06	0.05	0.009	0.049	<0.001	0.658	0.723	0.132	0.636
OTU84	*Flintibacter*	0.08	0.19	0.36	0.13	0.05	0.04	0.04	0.02	0.079	0.008	0.398	0.496	0.121	0.343
OTU78	*Anaerotruncus*	0.16	0.27	0.15	0.14	0.05	0.05	0.02	0.01	0.069	0.005	0.318	0.661	0.508	0.856
OTU92	*Anaerotruncus*	0.08	0.20	0.30	0.18	0.01	0.04	0.02	0.007	0.059	<0.001	0.278	0.918	0.111	0.340
OTU94	*Negativibacillus*	0.07	0.20	0.21	0.12	0.06	0.08	0.05	0.02	0.057	0.022	0.905	0.881	0.105	0.618
OTU83	*Anaerotruncus*	0.12	0.23	0.14	0.11	0.05	0.10	0.03	0.008	0.067	0.027	0.299	0.635	0.265	0.980
OTU72	*Eubacterium*	0.07	0.16	0.21	0.23	0.07	0.01	0.009	0.008	0.080	0.015	0.508	0.811	0.967	0.485
OTU80	*Acetivibrio*	0.02^{c}	0.05^{bcB}	0.20^{abA}	0.28^{a}	0.08^{bc}	0.04^{bcB}	0.07^{bc}	0.02^{c}	0.058	0.051	0.023	0.861	0.756	0.042
OTU93	*Lactobacillus*	0.09	0.04	0.07	0.11	0.11	0.05	0.11	0.17	0.033	0.146	0.065	0.946	0.039	0.867
OTU90	*Escherichia/Shigella*	0.06	0.05	0.05	0.10	0.10	0.10	0.12	0.13	0.026	0.013	0.250	0.701	0.439	0.878
OTU98	*Lactobacillus*	0.09	0.04	0.08	0.10	0.05	0.02	0.13	0.21	0.033	0.269	0.001	0.762	0.039	0.137
OTU97	*Oscillibacter*	0.11	0.20	0.17	0.10	0.05	0.02	0.05	0.004	0.040	<0.001	0.608	0.701	0.134	0.529
OTU106	*Lactobacillus*	0.08^{bc}	0.04^{c}	0.06^{bc}	0.08^{bc}	0.05^{bcC}	0.02^{c}	0.13^{abB}	0.20^{aA}	0.029	0.103	0.001	0.807	0.051	0.037
OTU112	*Enterococcus*	0.02	0.04	0.004	0.009	0.06	0.11	0.17	0.24	0.065	0.009	0.269	0.439	0.966	0.450
OTU124	*Eubacterium*	0.11	0.13	0.14	0.10	0.07	0.01	0.05	0.01	0.038	0.003	0.869	0.268	0.647	0.809
OTU101	*Lactobacillus*	0.02	0.10	0.01	0.008	0.10	0.02	0.20	0.18	0.054	0.028	0.307	0.897	0.913	0.077
OTU89	*Hespellia*	0.04	0.16	0.16	0.13	0.07	0.02	0.02	0.01	0.041	0.003	0.746	0.825	0.351	0.130
OTU118	*Lactobacillus*	0.08	0.03	0.08	0.07	0.04	0.01	0.12	0.16	0.029	0.347	0.003	0.526	0.198	0.149
OTU96	*Pseudoflavonifractor*	0.09	0.18	0.18	0.06	0.02	0.01	0.02	0.007	0.035	<0.001	0.760	0.624	0.033	0.262
OTU114	*Ethanoligenens*	0.05	0.14	0.22	0.11	0.02	0.02	0.02	0.004	0.041	<0.001	0.303	0.769	0.066	0.228
OTU108	*Lactobacillus*	0.05	0.04	0.05	0.07	0.08	0.08	0.10	0.09	0.023	0.037	0.334	0.865	0.862	0.884
OTU109	*Agathobaculum*	0.13	0.12	0.11	0.08	0.04	0.02	0.03	0.009	0.029	0.000	0.326	0.459	0.789	0.976
OTU58	*Anaerobacterium*	0.07	0.06	0.09	0.17	0.06	0.01	0.07	0.02	0.047	0.062	0.336	0.811	0.597	0.378
OTU136	*Lactobacillus*	0.07	0.03	0.08	0.06	0.01	0.03	0.14	0.10	0.032	0.629	0.005	0.325	0.604	0.323
OTU155	[*Clostridium*]	0.10	0.08	0.14	0.10	0.03	0.02	0.02	0.003	0.038	0.002	0.812	0.473	0.793	0.865

OTU143	*Lactobacillus*	0.06	0.03	0.07	0.05	0.01	0.03	0.13	0.08	0.029	0.537	0.011	0.332	0.423	0.301
OTU135	*Lactobacillus*	0.04	0.03	0.05	0.05	0.06	0.06	0.08	0.08	0.018	0.021	0.114	0.962	0.858	0.950
OTU113	*Comamonas*	0.10	0.08	0.02	0.16	0.003	0.008	0.005	0.06	0.039	0.011	0.622	0.105	0.064	0.719
OTU152	[*Clostridium*]	0.03	0.10	0.13	0.03	0.02	0.10	0.01	0.002	0.043	0.093	0.597	0.814	0.084	0.328
OTU158	*Escherichia/Shigella*	0.02	0.03	0.02	0.03	0.15	0.13	0.04	0.01	0.037	0.032	0.042	0.716	0.946	0.177
OTU123	*Escherichia/Shigella*	0.06	0.04	0.05	0.05	0.06	0.07	0.05	0.03	0.010	0.803	0.055	0.066	0.534	0.110

[1]Data are presented as least-square means ± pooled standard error of the mean (SEM). Low RFI *ad libitum* females, *n* = 7 per time point; Low RFI *ad libitum* males, *n* = 7 per time point; High RFI *ad libitum* females, *n* = 8 per time point; High RFI *ad libitum* males, *n* = 7 per time point; Low RFI restrictive females, *n* = 7 per time point; Low RFI restrictive males, *n* = 7 per time point; High RFI restrictive females, *n* = 7 per time point; High RFI restrictive males, *n* = 7 per time point.

[2]Taxonomic classification based on the National Center for Biotechnology Information (NCBI) nucleotide database (https://blast.ncbi.nlm.nih.gov/).

[3]TP, time point.

[4]Restr., restrictive feeding.

[a,b,c]Different superscripts within a row indicate significant difference (P ≤ 0.05).

[A,B,C,D]Different superscripts within a row indicate a tendency ($P \leq 0.10$).

Table S4. BLAST search results for selected operational taxonomic units (OTUs)[1,2]

OTU	Blast hit (16S NCBI database)	Accession Number	Percent idendity
OTU1	*Escherichia coli* strain JCM 1649	NR_112558.1	100
	Shigella flexneri strain ATCC 29903	NR_026331.1	100
OTU2	*Lactobacillus salivarius* strain HO 66	NR_028725.2	99
OTU3	*Turicibacter sanguinis* strain MOL361	NR_028816.1	99
OTU4	*Agathobaculum desmolans* strain ATCC 43058	NR_044644.2	98
OTU5	*Lactobacillus crispatus* strain DSM 20584	NR_119274.1	99
OTU6	*Escherichia coli* strain JCM 1649	NR_112558.1	99
	Shigella flexneri strain ATCC 29903	NR_026331.1	99
OTU7	*Lactobacillus johnsonii* strain CIP 103620	NR_117574.1	99
OTU8	*Lactobacillus crispatus* strain DSM 20584	NR_119274.1	99
OTU9	[*Clostridium*] *alkalicellulosi* strain Z-7026	NR_115345.1	87
OTU10	[*Clostridium*] *clariflavum* strain DSM 19732	NR_102987.1	87
OTU11	*Anaerobacterium chartisolvens* strain T-1-35	NR_125464.1	87
OTU13	*Acinetobacter bereziniae* strain ATCC 17924	NR_117625.1	99
OTU15	*Agathobaculum desmolans* strain ATCC 43058	NR_044644.2	98
OTU16	*Anaerobacterium chartisolvens* strain T-1-35	NR_125464.1	88
OTU18	*Klebsiella variicola* strain F2R9	NR_025635.1	99
OTU19	*Anaeromassilibacillus senegalensis* strain mt9	NR_144727.1	98
OTU20	*Lactobacillus reuteri* strain DSM 20016	NR_075036.1	99
OTU21	*Lactobacillus crispatus* strain DSM 20584	NR_119274.1	99
OTU22	*Turicibacter sanguinis* strain MOL361	NR_028816.1	99
OTU26	*Gracilibacter thermotolerans* strain JW/YJL-S1	NR_115693.1	87
OTU27	*Lactobacillus salivarius* strain HO 66	NR_028725.2	99
OTU30	*Lactobacillus crispatus* strain DSM 20584	NR_119274.1	99
OTU31	[*Ruminococcus*] *torques* strain VPI B2-51	NR_036777.1	97
OTU32	*Saccharofermentans acetigenes* strain P6	NR_115340.1	87
OTU33	*Escherichia coli* strain JCM 1649	NR_112558.1	99
	Shigella flexneri strain ATCC 29903	NR_026331.1	99
OTU34	*Turicibacter sanguinis* strain MOL361	NR_028816.1	92
OTU35	*Eisenbergiella massiliensis* strain AT11	NR_144731.1	95
OTU39	[*Clostridium*] *saccharolyticum* strain WM1	NR_102852.1	96
OTU42	*Lactobacillus crispatus* strain DSM 20584	NR_119274.1	99
OTU43	*Lactobacillus salivarius* strain HO 66	NR_028725.2	96
OTU44	*Turicibacter sanguinis* strain MOL361	NR_028816.1	99
OTU45	*Acinetobacter bereziniae* strain ATCC 17924	NR_117625.1	98
OTU47	*Lactobacillus salivarius* strain HO 66	NR_028725.2	97
OTU48	*Negativibacillus massiliensis* strain Marseille-P3213	NR_147378.1	93

OTU51	*Lactobacillus crispatus* strain DSM 20584	NR_119274.1	96
OTU52	[*Clostridium*] *leptum* strain DSM 753	NR_114789.1	97
OTU53	*Klebsiella oxytoca* strain NBRC 102593	NR_114152.1	99
OTU56	*Anaerobacterium chartisolvens* strain T-1-35	NR_125464.1	88
OTU58	*Anaerobacterium chartisolvens* strain T-1-35	NR_125464.1	88
OTU59	*Lactobacillus salivarius* strain HO 66	NR_028725.2	98
OTU60	[*Clostridium*] *leptum* strain DSM 753	NR_114789.1	94
OTU62	*Acinetobacter guillouiae* strain ATCC 11171	NR_117626.1	98
OTU67	*Lactobacillus crispatus* strain DSM 20584	NR_119274.1	97
OTU68	*Flintibacter butyricus* strain BLS21	NR_144611.1	98
OTU71	*Lactobacillus salivarius* strain HO 66	NR_028725.2	99
OTU72	*Eubacterium coprostanoligenes* strain HL	NR_104907.1	92
OTU73	*Klebsiella oxytoca* strain NBRC 102593	NR_114152.1	99
OTU74	*Agathobaculum desmolans* strain ATCC 43058	NR_044644.2	98
OTU75	*Eisenbergiella massiliensis* strain AT11	NR_144731.1	95
OTU76	*Lactobacillus salivarius* strain HO 66	NR_028725.2	95
OTU77	*Oscillibacter ruminantium* strain GH1	NR_118156.1	95
OTU78	*Anaerotruncus colihominis* strain WAL 14565	NR_027558.1	99
OTU79	[*Clostridium*] *leptum* strain DSM 753	NR_114789.1	95
OTU80	*Acetivibrio cellulolyticus* strain CD2	NR_025917.1	95
OTU82	*Klebsiella quasipneumoniae* subsp. *similipneumoniae* strain 07A044	NR_134063.1	98
OTU83	*Anaerotruncus colihominis* strain WAL 14565	NR_027558.1	99
OTU84	*Flintibacter butyricus* strain BLS21	NR_144611.1	98
OTU86	*Acinetobacter pittii* strain ATCC 19004	NR_117621.1	99
OTU89	*Hespellia porcina* strain PC80	NR_025206.1	96
OTU90	*Escherichia coli* strain JCM 1649	NR_112558.1	100
	Shigella flexneri strain ATCC 29903	NR_026331.1	100
OTU91	*Acinetobacter courvalinii* strain ANC 3623	NR_148843.1	95
OTU92	*Anaerotruncus colihominis* strain WAL 14565	NR_027558.1	92
OTU93	*Lactobacillus salivarius* strain HO 66	NR_028725.2	98
OTU94	*Negativibacillus massiliensis* strain Marseille-P3213	NR_147378.1	99
OTU95	*Escherichia coli* strain JCM 1649	NR_112558.1	92
	Shigella flexneri strain ATCC 29903	NR_026331.1	92
OTU96	*Pseudoflavonifractor phocaeensis* strain Marseille-P3064	NR_147370.1	96
OTU97	*Oscillibacter ruminantium* strain GH1	NR_118156.1	95
OTU98	*Lactobacillus crispatus* strain DSM 20584	NR_119274.1	99
OTU101	*Lactobacillus reuteri* strain DSM 20016	NR_075036.1	99
OTU103	*Turicibacter sanguinis* strain MOL361	NR_028816.1	91
OTU106	*Lactobacillus crispatus* strain DSM 20584	NR_119274.1	99
OTU108	*Lactobacillus salivarius* strain HO 66	NR_028725.2	90
OTU109	*Agathobaculum desmolans* strain ATCC 43058	NR_147371.1	91

OTU112	*Enterococcus villorum* strain NBRC 100699	NR_113935.1	99
OTU113	*Comamonas thiooxydans* strain S23	NR_115741.1	99
OTU114	*Ethanoligenens harbinense* strain YUAN-3	NR_074333.1	92
OTU118	*Lactobacillus crispatus* strain DSM 20584	NR_119274.1	99
OTU120	*Escherichia coli* strain JCM 1649	NR_112558.1	95
	Shigella flexneri strain ATCC 29903	NR_026331.1	95
OTU123	*Escherichia coli* strain JCM 1649	NR_112558.1	97
	Shigella flexneri strain ATCC 29903	NR_026331.1	97
OTU124	*Eubacterium coprostanoligenes* strain HL	NR_104907.1	95
OTU135	*Lactobacillus salivarius* HO 66	NR_028725.2	97
OTU136	*Lactobacillus crispatus* strain DSM 20584	NR_119274.1	99
OTU143	*Lactobacillus crispatus* strain DSM 20584	NR_119274.1	99
OTU152	[*Clostridium*] *celerecrescens* strain 18A	NR_026100.1	96
OTU155	[*Clostridium*] *lavalense* strain CCRI-9842	NR_044289.1	95
OTU158	*Escherichia coli* strain JCM 1649	NR_112558.1	99
	Shigella flexneri strain ATCC 29903	NR_026331.1	99

[1]BLAST search was performed for the most abundant OTUs in feces differently affected by time point, feeding level, or residual feed intake.

[2]National Center for Biotechnology Information (NCBI) nucleotide database (https://blast.ncbi.nlm.nih.gov/); last accessed 14/11/2017.
